# Nanotechnology-Based Drug Delivery Systems

**DOI:** 10.3390/pharmaceutics17070817

**Published:** 2025-06-24

**Authors:** Andrey N. Kuskov, Ekaterina V. Kukovyakina, Ekaterina N. Krasnoselskaya

**Affiliations:** Department of Technology of Chemical, Pharmaceutical and Cosmetic Substances, D. Mendeleev University of Chemical Technology of Russia, 125047 Moscow, Russia

Modern oncological and chronic inflammatory diseases require new approaches to drug therapy due to the limited effectiveness and several side effects of traditional drug preparations. Antitumor and anti-inflammatory drugs often have low selectivity of action, poor solubility, and bioavailability, which leads to insufficient drug concentration at the site of the disease and toxicity to healthy tissues [1]. To overcome these shortcomings, nanosystems have been developed for the delivery of biologically active substances, facilitating the targeted transport of these substances to a specific site in the organism and reducing drugs’ side effects. The encapsulation of biologically active substances in nano-scaled carriers allows for increasing the therapeutic index of drugs due to more targeted accumulation in the diseased organ and controlled release, resulting in high therapeutic concentrations of the drug in the lesion [2]. Nanoparticles up to 150−200 nm in size are able to overcome or bypass the biological barriers that hinder the delivery of traditional dosage forms [3]. Nanoparticles protect vulnerable drugs (e.g., enzymes, peptides, nucleic acids, etc.) from degradation by enzymes and rapid excretion by the kidneys, prolonging their circulation as well as promoting prolonged release of biologically active substances or, conversely, stimulus-sensitive “explosive” release. Finally, nanoparticles offer the possibility of multimodal therapy—combining several therapeutic and diagnostic functions in one carrier. Examples include theranostics (a nanoparticle simultaneously carries a drug and a contrast agent for tumor visualization) and combined nanopreparations (e.g., simultaneous delivery of two chemotherapy drugs or a combination of chemotherapy with a photothermal agent) [4]. Moreover, the surface of nanoparticles can be modified with ligands (antibodies, folates, etc.) for active targeting, i.e., directed binding to target cells, which further enhances targeted delivery. Thus, nanotechnology opens the way to personalized and highly precise disease therapy. Nanoparticles for drug delivery can be produced from a wide variety of materials, both organic and inorganic, which has led to the emergence of several important classes of nano-scaled carriers. Among the most developed are lipid nanoparticles, polymer nanoparticles, magnetic nanoparticles, biodegradable nanoparticles, and a number of other promising categories (e.g., metal and silicon nanostructures, dendrimers, and protein-based nanoparticles) [5,6,7]. Each of these classes has unique properties that determine its use in therapy and often specific limitations. Currently, many nano-sized formulations are already undergoing clinical trials or are used in the treatment of various diseases. These achievements confirm the significant potential of nanosystems for drug delivery in medicine.

The twelve articles included in the current Special Issue, entitled “Nanotechnology-Based Drug Delivery Systems”, present novel contributions and advances in research on nano-scaled carriers of biologically active substances for the treatment of various diseases and in vitro and in vivo investigations of such systems, as well as review articles outlining the current achievements in nano-drug delivery science.

Several articles in this Special Issue are focused on the development and study of nano-scaled systems for the delivery of anti-inflammatory agents: indomethacin (contribution 1) and the naturally occurring alkaloid berberine (contribution 2). Kuskov A.N. et al. present in vivo studies of nanoparticles based on amphiphilic derivatives of poly-N-vinylpyrrolidone with encapsulated indomethacin (contribution 1). Compared with free indomethacin, nanoparticles are characterized by controlled prolonged release of indomethacin, both in vitro and in vivo. Additionally, the prepared nanoparticles accumulated less in the liver and kidneys and improved pharmacokinetics. In addition, when administered subcutaneously in acute, subchronic, and chronic inflammation models, nanoparticles with indomethacin increased anti-inflammatory activity compared to free indomethacin and, when administered orally, were deemed safe for the gastrointestinal tract. For the treatment of rheumatoid arthritis, Mohammed H. Elkomy et al. obtained a gel based on chitosan-coated bilosomes loaded with berberine (BER-CTS-BLS) (contribution 2). The optimal composition of BER-CTS-BLS with a high desirability index was selected, which is characterized by the prolonged release of berberine in vitro, increased permeability through the skin without causing any irritation ex vivo, and an in vivo anti-inflammatory effect against carrageenan-induced inflammation. Thus, the gel based on BER-CTS-BLS can be a promising system for the transdermal delivery of biologically active substances to relieve inflammation in rheumatoid arthritis. Using a plant extract with anti-inflammatory properties in the synthesis of nanoparticles facilitates their application in the treatment of rheumatoid arthritis without the introduction of additional drugs (contribution 3). Anupama Singh et al. prepared silver nanoparticles using the plant extract of Commiphora mukul (gugul) as a reducing and stabilizing agent (G-AgNPs). G-AgNPs are colloidally stable in vitro and safe when administered orally in vivo, and their efficacy in the treatment of rheumatoid arthritis is comparable to that of the reference drug methotrexate.

To load drugs with various chemical compositions and from different pharmacotherapeutic groups into a proper carrier, selecting optimal conditions and developing a universal production method are crucial (contribution 4). Using soybean phosphatidylcholine, Elena G. Tikhonova et al. obtained nanoparticles that were spherical unilamellar vesicles. The particles were characterized by small sizes of ~30 nm; efficient drug incorporation (doxorubicin, indometacin, umifenovir, diclofenac, budesonid, prednisolone, and chlorin e6), reaching at least 92% efficacy; and prolonged release of active agents for over 72 h.

This Special Issue also includes several articles on the development of nano-carriers of antitumor agents used for the treatment of hepatocellular carcinoma (contribution 5), ovarian cancer (contribution 6), and breast cancer (contributions 7 and 8). Mohammed S. Saddik et al. obtained a nanocomposite comprising iron nanoparticles and copper nanoparticles with doxorubicin adsorbed on the surface (DOX-C@Fe@Cu NC) (contribution 5). Optimal conditions were determined for obtaining a composite with the highest degree of doxorubicin loading, which exhibited increased cytotoxicity compared to free doxorubicin and had a high percentage of late apoptosis. Ekaterina Sinitsyna et al. obtained nanoparticles based on block copolymers of poly(ethylene glycol) monomethyl ether (mPEG) and poly(D,L-lactic acid) (PLA)/poly(ε-caprolactone) (PCL) with dioxadet ([5-[[4,6-bis(aziridin-1-yl)-1,3,5-triazin-2-yl]amino]-2,2-dimethyl-1,3-dioxan-5-yl]methanol), a cytostatic drug from the group of triazine derivatives, loaded inside (contribution 6). Both types of nanoparticles (DOD/mPEG-b-PLA and DOD/mPEG-b-PCL) were stable for 3 weeks in water and PBS at +4 °C, and DOD/mPEG-b-PLA particles were also stable at +23 °C. The encapsulation of dioxadet in nanoparticles promoted the prolonged release of the drug, increasing its cytotoxicity on tumor cells while decreasing it on normal cells. Supusson Pengnam et al. prepared an andrographolide nanosuspension with a substituted dithiocarbamate moiety at position C12, stabilized by amphiphilic chitosan derivatives (3nAGN-NSC) (contribution 7). Notably, 3nAGN-NSC was more cytotoxic than unmodified andrographolide suspension, and together with the suppression of Mcl-1 expression by siRNA, it demonstrated a synergistic apoptotic effect at lower concentrations compared to individual administration of the components. Giovanni Smaldone et al. obtained nanogels based on Fmoc-FF peptides (Fmoc-Phe-Phe-OH, Nα-9-fluorenylmethoxycarbonyl-diphenylalanine) (contribution 8). Using caveolin-mediated endocytosis, these nanogels were able to penetrate cells overexpressing the caveolin-1 protein. It is worth noting that the higher the level of caveolin-1 expression in cells, the greater the cytotoxicity of the nanogel. Thus, nanogels based on Fmoc-FF peptides can selectively affect various cells.

Drug delivery to the desired site is achieved not only by drug encapsulation inside nano-carriers but also through the immobilization of active substances on their surface (contribution 9). For the treatment of abnormal lipid accumulation, Stavroula Zagkou et al. developed polylactide nanoparticles with Tat-Beclin peptide immobilized on the surface (NP T-B). Peptide-modified nanoparticles were able to induce autophagy for a long period with an enhanced effect and reduce the number of intracellular lipids at lower doses compared to free peptides, without exhibiting pronounced toxicity. In addition, autophagy-inducing nanoparticles were internalized and localized in endosomal, endolysosomal, and autolysosomal compartments, and they accumulated in the liver of both healthy and obese mice. Thus, NP T-B may be a promising therapeutic system for the treatment of non-alcoholic fatty liver diseases.

Lastly, the current Special Issue encompasses three reviews highlighting various nano-scaled drug delivery systems, their characteristics, and properties, as well as areas of their application (contributions 10−12). Ashwini T. et al. provide an overview of nanomaterials used in wound dressings, which protect wounds from infection, control bleeding, reduce pain and inflammation, and stimulate active repair of damaged tissues (contribution 10). They also reviewed various nanoparticles used in wound dressings to enhance cell proliferation, migration, and differentiation and presented the results of clinical trials of the final products. Their review concludes by presenting future perspectives and scope, highlighting the challenges associated with introducing nanomaterials to the market and expanding the range of their applications in tissue engineering and regenerative medicine. In the second review, Veronika Smagina et al. provide a comparative review of nano-sized hydrogel systems based on polymers of various origins that can be used for targeted drug delivery in the treatment of a wide range of diseases (contribution 11). The authors compared the main characteristics of hydrogels for biomedicine, developed in 2021-2022: cytotoxic activity and the mechanism and rate of drug release from hydrogels. The third review by Jaeseong Lee et al. focuses on various nanoparticles that deliver drugs to metastatic lymph nodes and their in vivo antimetastatic efficacy (contribution 12). They also present data on the application of such particles in clinical practice as contrast agents for sentinel lymph node identification.

The development of nano-scaled systems for drug delivery is a novel promising approach to disease therapy that has raised interest in the scientific community worldwide. We thank all the authors of this Special Issue for their valuable contribution and wish them success in their future research.
